# PTPN1 is a prognostic biomarker related to cancer immunity and drug sensitivity: from pan-cancer analysis to validation in breast cancer

**DOI:** 10.3389/fimmu.2023.1232047

**Published:** 2023-10-23

**Authors:** Ruijun Zhao, Shuanglong Chen, Weiheng Cui, Chaoyu Xie, Aiping Zhang, Li Yang, Hongmei Dong

**Affiliations:** ^1^ Department of Breast Surgery, The Third Hospital of Nanchang, Nanchang, China; ^2^ Department of General Surgery, The First Affiliated Hospital of Jinan University, Jinan University, Guangzhou, China; ^3^ Institute of Precision Cancer Medicine and Pathology, and Department of Pathology, School of Medicine, Jinan University, Guangzhou, China; ^4^ Department of Breast Surgery, Suichuan County Maternal and Child Health Care Hospital, Jian, China; ^5^ Department of Breast Surgery, Nancheng County Hospital of Traditional Chinese Medicine, Fuzhou, China

**Keywords:** pan-cancer, PTPN1, prognosis, tumor microenvironment, immunotherapy, drug sensitivity

## Abstract

**Background:**

Protein tyrosine phosphatase non-receptor type 1 (PTPN1), a member of the protein tyrosine phosphatase superfamily, has been identified as an oncogene and therapeutic target in various cancers. However, its precise role in determining the prognosis of human cancer and immunological responses remains elusive. This study investigated the relationship between PTPN1 expression and clinical outcomes, immune infiltration, and drug sensitivity in human cancers, which will improve understanding regarding its prognostic value and immunological role in pan-cancer.

**Methods:**

The PTPN1 expression profile was obtained from The Cancer Genome Atlas and Cancer Cell Line Encyclopedia databases. Kaplan-Meier, univariate Cox regression, and time-dependent receiver operating characteristic curve analyses were utilized to clarify the relationship between PTPN1 expression and the prognosis of pan-cancer patients. The relationships between PTPN1 expression and the presence of tumor-infiltrated immune cells were analyzed using Estimation of Stromal and Immune cells in Malignant Tumor tissues using Expression data and Tumor Immune Estimation Resource. The cell counting kit-8 (CCK-8) assay was performed to examine the effects of PTPN1 level on the sensitivity of breast cancer cells to paclitaxel. Immunohistochemistry and immunoblotting were used to investigate the relationship between PTPN1 expression, immune cell infiltration, and immune checkpoint gene expression in human breast cancer tissues and a mouse xenograft model.

**Results:**

The pan-cancer analysis revealed that PTPN1 was frequently up-regulated in various cancers. High PTPN1 expression was associated with poor prognosis in most cancers. Furthermore, PTPN1 expression correlated highly with the presence of tumor-infiltrating immune cells and the expression of immune checkpoint pathway marker genes in different cancers. Furthermore, PTPN1 significantly predicted the prognosis for patients undergoing immunotherapy. The results of the CCK-8 viability assay revealed that PTPN1 knockdown increased the sensitivity of MDA-MB-231 and MCF-7 cells to paclitaxel. Finally, our results demonstrated that PTPN1 was associated with immune infiltration and immune checkpoint gene expression in breast cancer.

**Conclusion:**

PTPN1 was overexpressed in multiple cancer types and correlated with the clinical outcome and tumor immunity, suggesting it could be a valuable potential prognostic and immunological biomarker for pan-cancer.

## Introduction

Cancer is the major cause of morbidity and mortality worldwide and a significant barrier to extending life expectancy because of the increasing number of newly diagnosed cases (1). Studies show that the tumor microenvironment (TME) is important in regulating cancer progression and clinical outcomes (2, 3). The TME primarily comprises immune cells surrounding the tumor, which can contribute to anti-tumor or tumor-promoting functions and provide opportunities for cancer immunotherapy (4). Increased levels of tumor-infiltrating CD8^+^ T cells are generally associated with favorable prognosis (5–7), whereas M2-like tumor-associated macrophages (TAMs) and T regulatory (Treg) cells have been widely recognized as negative prognostic indicators in cancers (8–10). Immunotherapy, particularly the use of immune checkpoint inhibitors (ICIs), has emerged as the dominant cancer treatment strategy in recent years, demonstrating remarkable clinical benefits in patients with different types of cancer (11). Despite the remarkable clinical efficacy of ICIs, only 20% of patients benefit from a long-term response to ICI therapies (12). Therefore, the development of therapeutic approaches for improving the clinical outcomes of cancer patients and identifying novel biomarkers for optimizing the selection of immunotherapy are urgently required.

Protein tyrosine phosphatases (PTPs), a large family of signaling enzymes, work in tandem with protein tyrosine kinases (PTKs) to regulate protein tyrosine phosphorylation (13). PTPs are classified into four distinct families based on the amino acid sequences of their catalytic domains (14). Non-receptor PTPs (PTPNs), members of the class I cysteine PTP family, have been implicated in various pathophysiological processes, including glucose homeostasis, inflammatory response, and immune response (15). PTPN1 (also known as PTP1B), a prominent member of the PTPNs, has been identified as a negative modulator of insulin and leptin receptor signaling (16). Recent evidence demonstrated that the role of PTPN1 varies with tumor types and can act as both a tumor suppressor and tumor promoter (17). In addition, PTPN1 plays a critical role in immune regulation and may be a promising target for immunotherapy. Previous studies have revealed that PTPN1 inhibits the activation of B cells and macrophages via B cell receptor signaling and macrophage-colony stimulating factor 1 signaling, respectively (18, 19). Notably, T cell anti-tumor immunity and dendritic cell-based immunotherapy can be improved by inhibiting PTPN1 (20, 21). Nonetheless, current studies on PTPN1 are limited to a few tumor types, and the prognostic value and immunity-associated role of PTPN1 in human tumors is still elusive and warrants further investigation.

In this study, we comprehensively investigated the impact of PTPN1 on the prognosis and immunotherapeutic predictive value in human cancers using multiple databases. Collectively, our results suggest that PTPN1 may be a novel promising prognostic biomarker and therapeutic target for pan-cancer patients.

## Methods and materials

### Data acquisition

The RNA-sequencing (RNA-seq) data in the form of log 2 (FPKM + 0.1) and the corresponding clinical data of pan-cancer patients from The Cancer Genome Atlas (TCGA) were acquired from the UCSC Xena browser (https://xenabrowser.net/), which included data of patients with adrenocortical carcinoma (ACC), bladder cancer (BLCA), breast carcinoma (BRCA), cervical cancer (CESC), cholangiocarcinoma (CHOL), colorectal cancer (COAD), esophageal cancer (ESCA), glioblastoma (GBM), head and neck squamous cell carcinoma (HNSC), kidney chromophobe (KICH), kidney renal clear cell carcinoma (KIRC), kidney renal papillary cell carcinoma (KIRP), low grade glioma (LGG), liver hepatocellular carcinoma (LIHC), lung adenocarcinoma (LUAD), lung squamous cell carcinoma (LUSC), mesothelioma (MESO), ovarian serous cystadenocarcinoma (OV), pancreatic adenocarcinoma (PAAD), pheochromocytoma and paraganglioma (PCPG), prostate cancer (PRAD), rectal cancer (READ), sarcoma (SARC), skin cutaneous melanoma (SKCM), stomach adenocarcinoma (STAD), testicular cancer (TGCT), thyroid carcinoma (THCA), thymoma (THYM), uterine corpus endometrial carcinoma (UCEC), uterine carcinosarcoma (UCS), and ocular melanomas (UVM). Furthermore, the RNA-seq data were transformed into log 2 (TPM + 1) for all subsequent analyses. The expression matrix of pan-cancer cell lines was obtained from the Cancer Cell Line Encyclopedia (CCLE database (https://portals.broadinstitute.org/ccle). The immunofluorescence staining images of PTPN1 in two human cancer cell lines (A-431 and U-251MG) were downloaded from the Human Protein Atlas (HPA; www.proteinatlas.org) online platform.

### Prognostic analysis of PTPN1 in pan-cancer samples

Univariate Cox regression analysis and Kaplan-Meier survival analysis were used to investigate the prognostic value of PTPN1 with respect to overall survival (OS) and disease-specific survival (DSS) of patients with 33 types of cancer in TCGA. The R packages, “timeROC” (22) and “ggplot2” (*Elegant Graphics for Data Analysis” by Hadley Wickham;* http://ggplot2.org), were used to assess and visualize the predictive performance of PTPN1, respectively. Furthermore, the Kaplan-Meier Plotter online website (http://kmplot.com/analysis/) was also used to investigate the prognostic value of PTPN1 in pan-cancer samples.

### Analysis of the relationship between PTPN1 expression and the immune landscape pan-cancer

The Tumor Immune System Interaction Database (TISIDB) (http://cis.hku.hk/TISIDB/index.php) (23) was used to investigate the relationships between PTPN1 expression and immune or molecular subtypes of various types of cancer. The relationship between PTPN1 and the immune landscape of pan-cancer samples was analyzed using the Estimation of Stromal and Immune cells in Malignant Tumor tissues using Expression data (ESTIMATE) and Tumor Immune Estimation Resource (TIMER) (https://cistrome.shinyapps.io/timer/) (24). For each patient, the stromal score and immune score were generated using the R package “ESTIMATE” (https://R-Forge.R-project.org/projects/estimate/).

### Analysis of the relationship between PTPN1 expression and tumor mutational burden or microsatellite instability in pan-cancer samples

Sixty marker genes of immune checkpoint pathways (inhibitory genes, n = 24; stimulatory genes, n = 36) were collected, and the Spearman correlation coefficient between the expression of PTPN1 and that of these genes was calculated using SangerBox (http://sangerbox.com/). Correlation analysis between the PTPN1 expression and TMB or MSI was performed using Spearman’s method, which was visualized in the form of radar charts using the R package “fmsb” (M. Nakazawa, https://CRAN.R-project.org/package=fmsb). To further investigate the relationship between PTPN1 and immunotherapy, the IMvigor210 (298 patients with urological cancer), GSE78220 (27 patients with melanoma), and GSE91061 cohorts (49 patients with melanoma) were obtained, and the corresponding expression profiles were transformed into log 2 (TPM + 1).

### Drug sensitivity analysis

Gene expression in the NCI-60 cancer cell line and drug sensitivity data were obtained from the CellMiner website (https://discover.nci.nih.gov/cellminer), and the potential relationship between PTPN1 expression and drug sensitivity in cancer cell lines was investigated. In addition, The Genomics of Drug Sensitivity in Cancer (GDSC) database was also used to investigate the correlation between PTPN1 expression and anticancer drug sensitivity.

### Cell culture

All cell lines were obtained from the American Type Culture Collection (ATCC, Manassas, VA, USA). MDA-MB-231, MCF-7, and 4T1 cells were cultured in Dulbecco’s modified Eagle’s medium (DMEM)/F12 (Gibco, Invitrogen, Carlsbad, CA, USA) supplemented with 10% fetal bovine serum (FBS) at 37°C in a humidified incubator containing 5% CO_2_.

### Cell viability assay

Cell viability was examined using the cell counting kit-8 (CCK-8) assay. The MDA-MB-231 and MCF-7 cells were seeded at a density of 5 × 10^3^ cells per well in a 96-well plate and incubated at 37°C. After culturing overnight, the cells were treated with various concentrations of paclitaxel (0, 5, 10, 15, 20, 25, and 30 µM) and incubated for 72 h. Then, 10 μL CCK-8 reagent was added to the culture medium and incubated at 37°C for 2 h. The absorbance was measured at 450 nm using a microplate reader (Thermo Fisher Scientific).

### Quantitative reverse transcription polymerase chain reaction

Total RNA was extracted from the cells using TRIzol (Invitrogen, Waltham, MA, USA) according to the manufacturer’s instructions. RNA (4 μg) was reverse transcribed using oligo dT primer (Invitrogen) and M-MLV reverse transcriptase (Invitrogen). Subsequently, cDNA amplification was performed using the SYBR Green PCR amplification kit (Invitrogen) and the ABI 7500 real-time PCR system (Applied Biosystems, Waltham, MA, USA). All reactions were run in triplicate and normalized to GAPDH expression.

### Immunoblotting

Total proteins were extracted from cells using ice-cold radioimmunoprecipitation assay cell lysis buffer supplemented with protease inhibitors (Sigma Aldrich, St. Louis, MO, USA). Equal amounts of protein lysates were separated using 12% sodium dodecyl sulfate-polyacrylamide gel electrophoresis. The isolated proteins were then transferred from the gel to a polyvinylidene fluoride (PVDF) membrane. The PVDF membranes were blocked with 5% skimmed milk in Tris-buffered saline containing 0.1% Tween-20 (TBST) and incubated for 2 h at room temperature (RT). Subsequently, the membranes were incubated with the following primary antibodies overnight at 4°C: anti-PTPN1 (Cat. No. #5311; Cell Signaling Technology, Beverly, MA, USA); anti-β-actin (Cat. No. #4967; Cell Signaling Technology). Then, the membranes were incubated with horseradish enzyme-labeled secondary antibody for 1 h at RT. Finally, the enhanced chemiluminescence kit was used to detect protein signals.

### Patients

Patients with breast cancer (n = 60) who underwent surgical treatment at the Department of Breast Surgery, The Third Hospital of Nanchang from December 2019 to September 2020 were included in the study. All patients underwent surgical intervention as the primary treatment, followed by adjuvant radiotherapy, chemotherapy, or hormone therapy. The mean age of the patients was 50 years (range: 22–78 years). The clinical research protocols of this study were reviewed and approved by the Ethics Committee of The Third Hospital of Nanchang (IRB serial number: #K-ky2023049). Written informed consent was obtained from the patients in accordance with the principles expressed in the Declaration of Helsinki.

### Immunohistochemistry

In brief, 4 µm sections from representative breast cancer tumor tissue were excised from formalin-fixed paraffin-embedded specimens and subjected to deparaffinization, rehydration, blocking of endogenous peroxidase activity, and antigen retrieval. The following primary antibodies were used: anti-PTPN1 (Cat# ab252928; Abcam, Cambridge, UK), anti-CD8 (Cat# ab101500; Abcam), anti-PD-L1 (Cat# 66248-1-Ig; Proteintech Group Inc, Chicago, IL, USA), CD68 (Cat# ab955; Abcam), and CD163 (Cat# ab182422; Abcam). The samples were incubated with primary antibodies overnight at 4°C. Then, the sections were incubated with horseradish peroxidase-conjugated secondary antibodies at RT for 1 h, followed by color development using the 3,3′-diaminobenzidine substrate (DAKO, Glostrup, Denmark). The nuclei were counterstained using hematoxylin. The percentage of PTPN1 expression in the tumor cells was scored using the following scales: 0, negative; 1, ≤10%; 2, 11–50%; 3, 51–75%; 4, >75%. The staining intensity was scored using the following scales: 1, weak staining; 2, moderate staining; 3, strong staining. The percentage (P) and intensity (I) of cytoplasmic expression were multiplied to generate a numerical score (S = P*I).

### Immunofluorescence

For immunofluorescence analysis, xenograft tumor specimens were fixed using 4% formalin and embedded in paraffin, followed by deparaffinization, rehydration, endogenous peroxidase blocking, antigen retrieval, blocking with blocking buffer, and incubation with primary antibodies overnight at 4°C. The following primary antibodies were used: anti-CD8 (Cat# ab217344; Abcam), anti-PD-L1 (Cat# 66248-1-Ig; Proteintech Group Inc), F4/80 (Cat# ab16911; Abcam), and CD163 (Cat# ab182422; Abcam). The sections were washed with washing buffer and incubated with Alexa-Fluor-594 (red) and Alexa-Fluor-488 (green)-conjugated secondary antibodies for 1 h at RT. The sections were stained with 4′,6-diamidino-2-phenylindole before mounting and imaging on Cytation 5 cell imaging multi-mode reader (BioTek, USA). Semi-quantitative analysis was performed by determining the percentage of positive-stained cells using the ImageJ software (v1.8.0q, http://rsb.info.nih.gov/ij).

### Statistical analysis

All statistical analyses and visualizations were performed using the R version 4.1.0 software (R Foundation for Statistical Computing, Vienna, Austria). Data comparisons with normal distributions between two groups were performed using the Student’s *t*-test and those among more than two groups using one-way analysis of variance (ANOVA) with *post hoc* intergroup comparisons. The R packages “survival” (https://CRAN.R-project.org/package=survival) and “survminer” (https://CRAN.R-project.org/package=survminer) were used to perform univariate Cox proportional hazard regression and Kaplan-Meier survival analyses. The log-rank test was used to calculate statistical differences between curves in the Kaplan-Meier survival analysis. The GeneMANIA website (https://genemania.org/) was used to generate the protein-protein interaction (PPI) network. In addition, the R package, “clusterProfiler” (25), was used to perform Gene Ontology (GO) and Kyoto Gene and Genomic Encyclopedia (KEGG) enrichment analysis. The correlation between PTPN1 and immune checkpoint gene expression was evaluated using Pearson’s correlation test. *P* values less than 0.05 were considered statistically significant.

## Results

### Pan-cancer analysis of PTPN1 expression in normal and cancerous tissues

**Figure 1:**
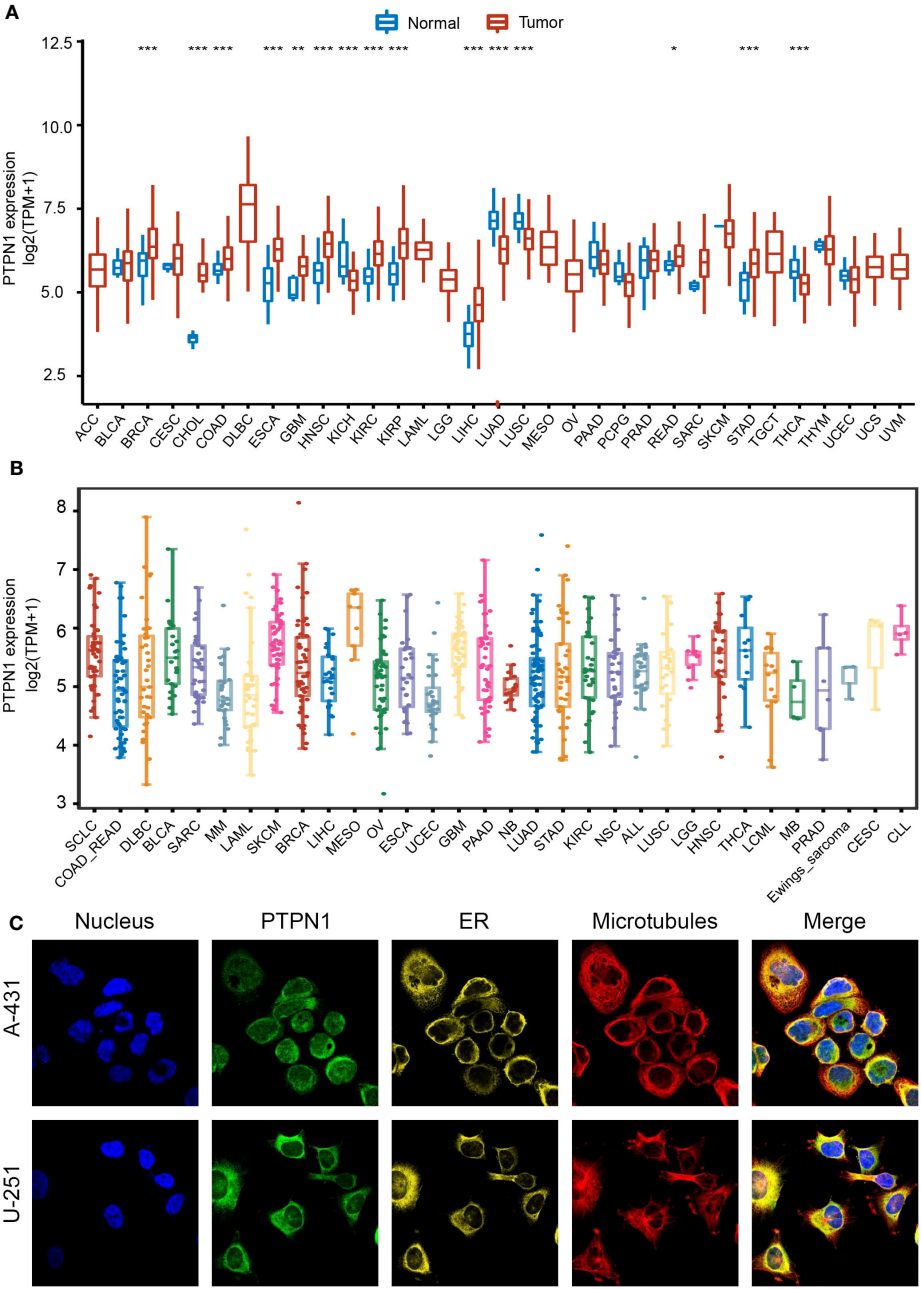
Expression landscape of PTPN1 in pan-cancer. **(A)** Distribution of PTPN1 expression in various cancer types from TCGA database. **(B)** PTPN1 expression level in various cancer cell lines from the CCLE database. **(C)** Representative images of immunofluorescence staining of PTPN1 protein in the nucleus, endoplasmic reticulum (ER), and microtubules in A-431 and U-251 cell lines from the HPA database. *P < 0.05, **P < 0.01, ***P < 0.001 by Student’s t-test.

To adequately investigate the distribution of PTPN1 expression between cancer and normal tissues, we examined *PTPN1* mRNA expression in the 33 cancer types from TCGA. The results showed that *PTPN1* expression was significantly higher in BRCA, CHOL, COAD, ESCA, GBM, HNSC, KIRC, KIRP, LIHC, READ, and STAD (**Figure 1A**), while it was lower in KICH, LUAD, LUSC, and THCA than that in the corresponding normal tissues (**Figure 1A**). Furthermore, the distribution of PTPN1 expression in various cancer cell lines was investigated using the CCLE database, and PTPN1 was found to be widely expressed in almost all types of cancer cell lines (**Figure 1B**). Additionally, the immunofluorescence staining images of two cancer cell lines (human epidermoid carcinoma cell line A-431 and malignant human glioma cell lines U-251) obtained from the HPA database showed that PTPN1 was mainly localized in the endoplasmic reticulum (ER) (**Figure 1C**). Collectively, PTPN1 expression in different tumors varied, leading to its dual role in tumorigenesis.

### Analysis of the prognostic value of PTPN1 in pan-cancer

**Figure 2:**
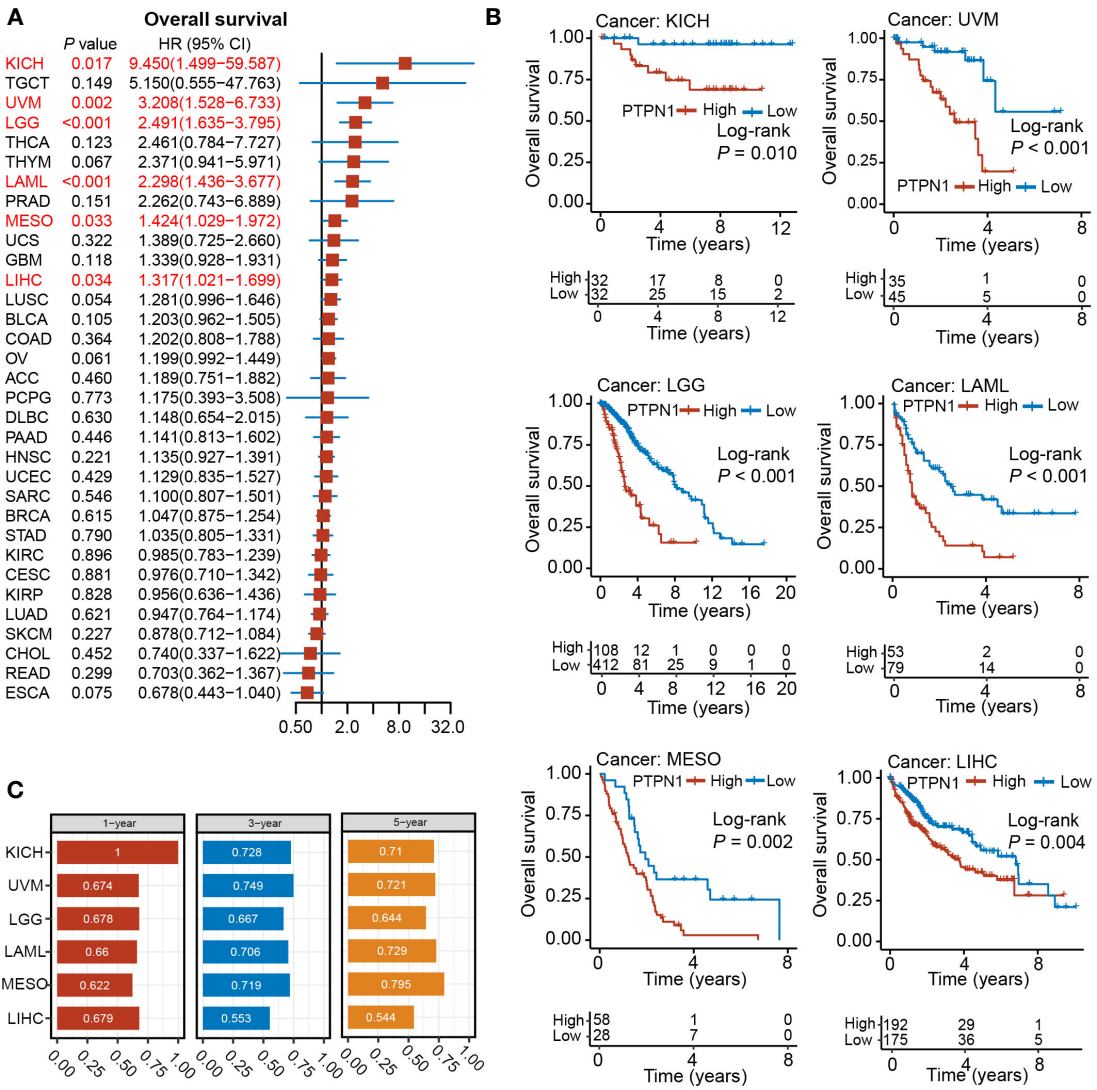
Relationship between PTPN1 expression and OS in pan-cancer. **(A)** The relationship between PTPN1 expression and OS in the cancer types indicated was analyzed using univariate Cox regression analyses. **(B)** The impact of PTPN1 on OS in the cancer types indicated was assessed using Kaplan-Meier survival curves. **(C)** Time-dependent ROC curve analysis was used to assess the performance of PTPN1 in predicting 1-, 3-, and 5-year OS for the cancer types indicated.

To further investigate the clinical significance of PTPN1 expression in human cancers, its prognostic value with respect to OS and DSS was analyzed using univariate Cox regression and Kaplan-Meier (log-rank test) analyses. As shown in **Supplementary Figure S1A**, PTPN1 expression correlated highly with the prognosis of most cancers except for ACC, CHOL, COAD, DLBC, PCGP, PRAD, READ, STAD, TGCT, UCEC, and UCS. Patients of each cancer type were divided into two groups based on the best cut-off value for PTPN1 expression: high and low expression. Using a log-rank test, we found that *PTPN1* mRNA expression was significantly associated with OS of patients with 19 cancer types, namely, BLCA, CESC, ESCA, GBM, HNSC, KICH, KIRC, LAML, LGG, LIHC, LUSC, MESO, OV, PAAD, SARC, SKCM, THCA, THYM, and UVM (**Supplementary Figure S1**). Univariate Cox regression analyses revealed that PTPN1 expression correlated significantly with OS of patients with six types of cancer, including KICH, LAML, LGG, LIHC, MESO, and UVM (**Figure 2A**). It is noteworthy that PTPN1 was a risk factor for OS of patients with KICH, UVM, LGG, LAML, MESO, and LIHC and that it correlated significantly with poor prognosis (**Figures 2A, B**). Furthermore, the time-dependent receiver operating characteristics (ROC) analysis revealed that PTPN1 had good predictive performance for 1-year, 3-year, and 5-year OS in patients with the six types of cancer mentioned above (**Figure 2C**).

**Figure 3:**
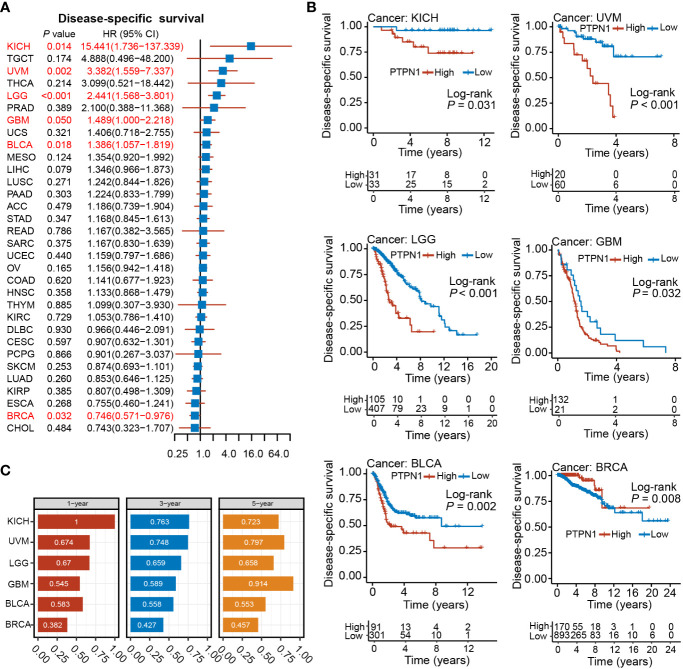
Relationship between PTPN1 expression and DSS in pan-cancer. **(A)** The relationship between PTPN1 expression and DSS in the cancer types indicated was analyzed using univariate Cox regression analysis. **(B)** Kaplan-Meier survival curves showing the impact of PTPN1 on DSS in the cancer types indicated. **(C)** The performance of PTPN1 in predicting the 1-, 3-, and 5-year DSS for the cancer types indicated was evaluated using time-dependent ROC curve analysis.

The log-rank test showed that patients with high PTPN1 expression had significantly worse DSS in BLCA, GBM, HNSC, KICH, KIRC, LGG, LIHC, MESO, OV, PAAD, SARC, THCA, and UVM, while a significantly longer DSS was observed in BRCA, CESC, KIRP, LUAD, and SKCM (**Supplementary Figure S1**). Univariate cox regression analysis revealed that PTPN1 was a risk factor for DSS in patients with BLCA, GBM, KICH, LGG, and UVM, while it was a protective factor for DSS in patients with BRCA (**Figure 3A**). Furthermore, PTPN1 was found to be a significant prognostic factor for DSS of patients with KICH, UVM, LGG, GBM, BLCA, and BRCA using the Kaplan-Meier (log-rank test) method (**Figure 3B**). In addition, PTPN1 showed good predictive performance for 1-year, 3-year, and 5-year DSS in patients with the cancer types mentioned above, as observed using time-dependent ROC analysis (**Figure 3C**). The calibration curves further revealed that the nomogram performed well in predicting the survival of cancer patients (**Supplementary Figures S2-5**). The C-index of the nomogram in BRCA, BLCA, KICH, and UVM cases were 0.747, 0.679, 0.948, and 0.759, respectively, which showed that the prediction model had good discrimination capacity.

Furthermore, we explored the prognostic value of PTPN1 in several types of cancer in the Kaplan-Meier Plotter database and found that PTPN1 was significantly associated with poor OS and post-progression survival (PPS) of patients with ovarian cancer, as well as poor OS, first progression (FP), and PPS of patients with lung cancer, and poor OS and PPS of patients with gastric cancer. On the contrary, increased PTPN1 was associated with significantly better OS, distant metastasis-free survival (DMSF), PPS, and recurrence-free survival (RFS) in patients with breast cancer, significantly better progression-free survival (PFS) in patients with ovarian cancer, and with a significantly better PPS in patients with gastric cancer (**Supplementary Figure S6**; *P* < 0.05 for all). Collectively, these results suggested a remarkable role for PTPN1 in predicting OS and DSS across multiple cancer types.

### PTPN1 expression was related to immune and molecular subtypes in human cancers

**Figure 4:**
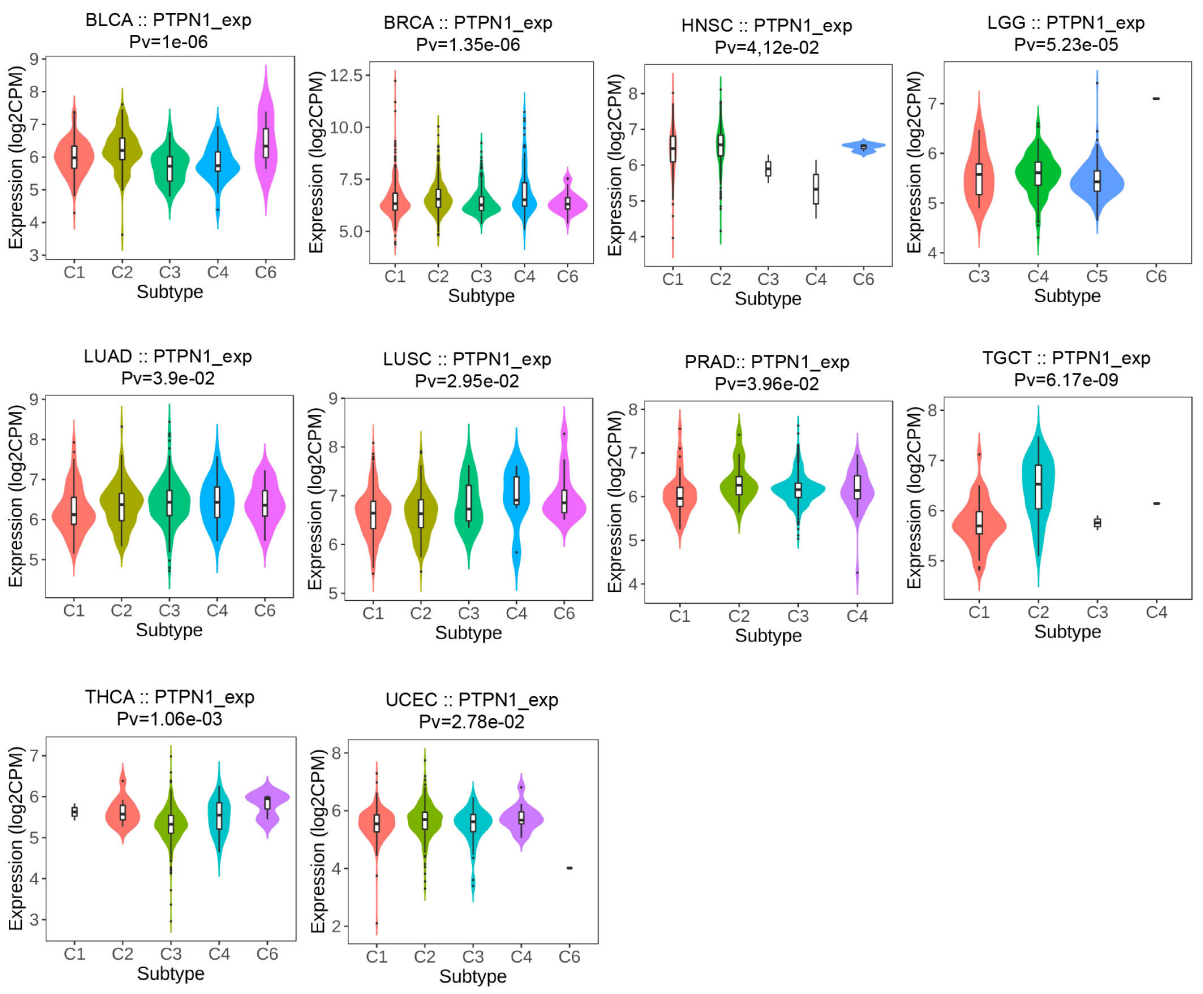
Relationship between PTPN1 expression and immune subtypes in cancers.

The TISIDB online website was further used to detect the relevance of PTPN1 for multiple immune subtypes (C1: wound healing, C2: IFN-gamma dominant, C3: inflammatory, C4: lymphocyte-depleted, C5: immunologically quiet, and C6: TGF-β dominant) of different cancer types. The results showed that PTPN1 expression was associated with various immune subtypes in patients with BLCA, BRCA, HNSC, LGG, LUAD, LUSC, PRAD, TGCT, THCA, and UCEC (**Figure 4**; *P* < 0.05 for all). Using BRCA as an example, we found that PTPN1 was highly expressed in the C4 type, followed by that in the C2 type. A significant link between PTPN1 expression and various cancer molecular subtypes was found in ACC, BRCA, COAD, ESCA, HNSC, LGG, LUSC, OV, PRAD, READ, STAD, and UCEC (**Supplementary Figure S7**; *P* < 0.05 for all). To summarize, PTPN1 expression varied between immune subtypes and molecular subtypes of various human cancers.

### Role of PTPN1 in determining the TME of pan-cancer

**Figure 5:**
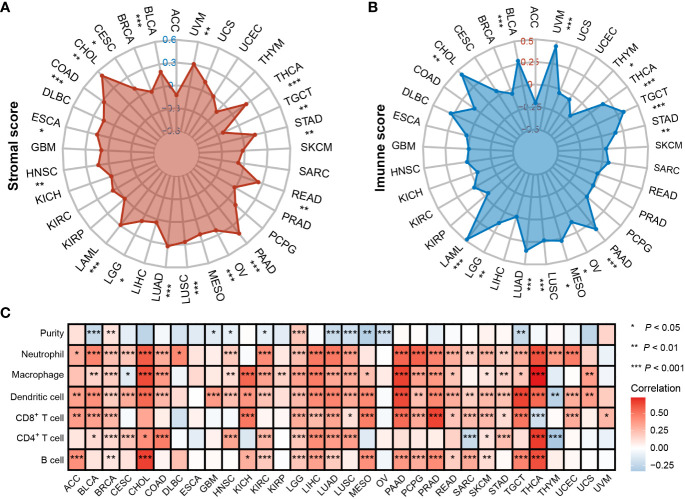
Correlation between PTPN1 level and immune infiltrates in various cancers. **(A, B)** Radar charts show the relationship between PTPN1 expression and stromal score **(A)** and immune score **(B)** in pan-cancer. **(C)** The heat map shows the correlation between the expression level of PTPN1 and the presence of immune infiltrates in pan-cancer.

To understand whether PTPN1 was crucial in determining the TME of human cancers, we used the ESTIMATE and TIMER 2.0 databases to examine the relationship between PTPN1 expression and immune cell infiltration in various cancers. The ESTIMATE results showed that PTPN1 expression was related to stromal score in the following cancers: BLCA, CESC, CHOL, COAD, ESCA, HNSC, LAML, LGG, LUAD, LUSC, OV, PAAD, READ, STAD, TGCT, THCA, and UVM (**Figure 5A**; *P* < 0.05 for all). In multiple cancer types, including BLCA, CHOL, LAML, LGG, LUAD, LUSC, MESO, OV, PAAD, STAD, TGCT, THCA, THYM, and UVM (**Figure 5B**; *P* < 0.05 for all), PTPN1 maintained a close relationship with the immune score.

Next, the TIMER 2.0 database was used to assess the infiltration levels of six immune cell types in each patient in TCGA pan-cancer cohort. PTPN1 expression showed a significant positive correlation with most types of immune cells, including B cells, CD4^+^ T cells, CD8^+^ T cells, neutrophils, macrophages, and dendritic cells in most cancer types, especially in breast cancer (**Figure 5C**; *P* < 0.05 for all). Our findings suggest that PTPN1 may play an important role in regulating immune cell infiltration in various tumors.

### Correlation of PTPN1 expression with immune checkpoint-related genes in pan-cancer

**Figure 6:**
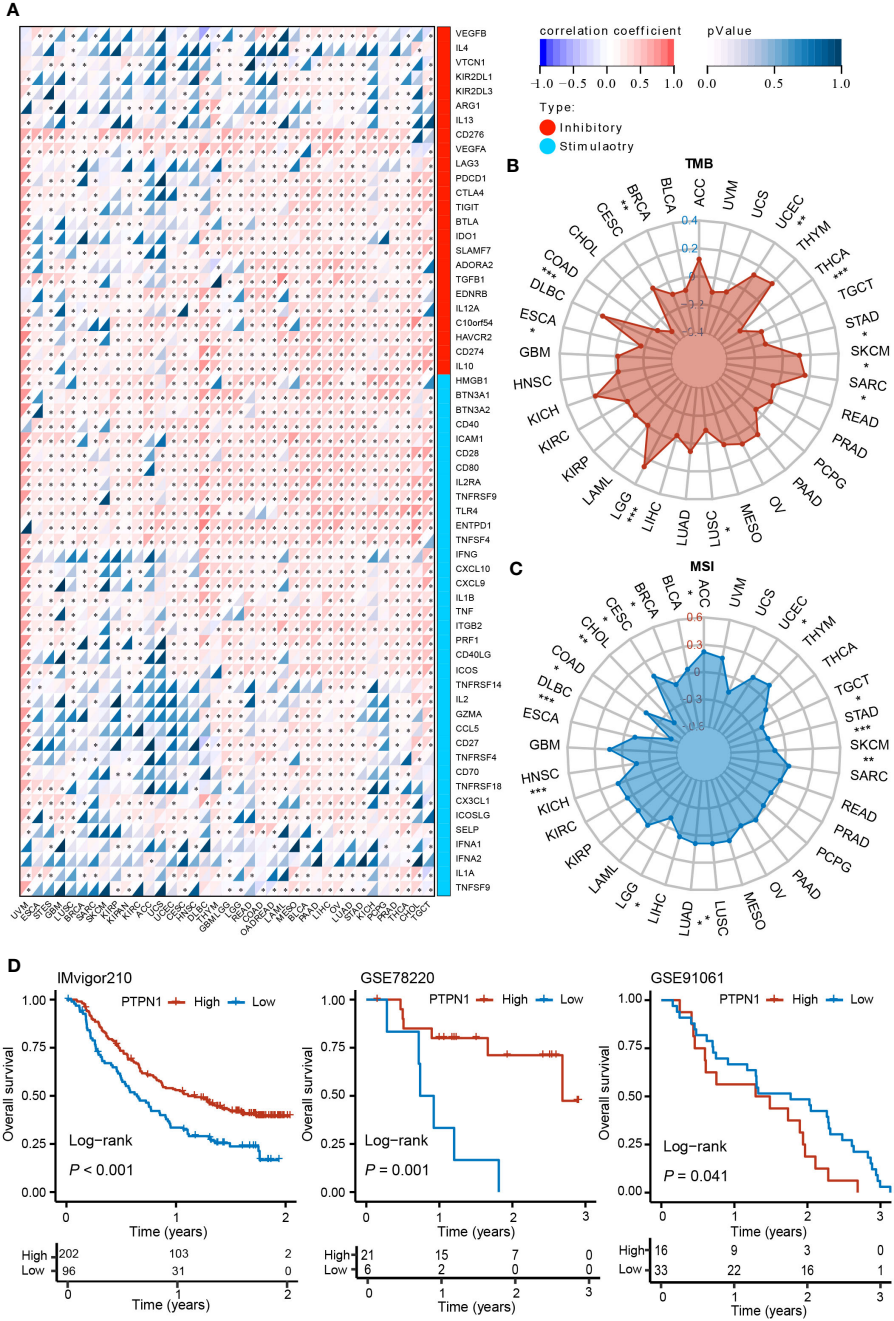
Correlation between PTPN1 level and expression of immune checkpoint-related genes in various cancers. **(A)** The heat map shows the correlation between the expression levels of PTPN1 and immune checkpoint genes in pan-cancer. **(B, C)** Radar charts show the relationship between PTPN1 expression and TMB **(B)** and MSI **(C)** in pan-cancer. **(D)** Kaplan-Meier curve showing the impact of PTPN1 on OS of patients from the IMvigor210, GSE78220, and GSE91061 cohorts. ^*^ *P* < 0.05; ^**^ *P* < 0.01; ^***^ *P* < 0.001.

Previous studies have shown that immune checkpoint-related genes considerably influence immune cell infiltration and immunotherapy (26). Therefore, we investigated the potential role of PTPN1 in immunotherapy by examining the relationship between the expression levels of PTPN1 and immune checkpoint-related genes in human cancers. The findings revealed that PTPN1 expression was related to the majority of immune checkpoint-related genes, particularly in PAAD, LIHC, LUAD, OV, and UVM (**Figure 6A**; *P* < 0.05 for all).

We further found that PTPN1 expression correlated positively with TMB in patients with LGG, SARC, SKCM, and UCEC (*P* < 0.05 for all), while it correlated negatively with TMB in patients with BRCA, COAD, ESCA, LUSC, STAD, and THCA (**Figure 6B**; *P* < 0.05 for all). Moreover, we found that PTPN1 correlated positively with MSI in patients with ACC, CESC, LUAD, LUSC, and UCEC (**Figure 6C**; *P* < 0.05 for all), while it correlated negatively with MSI in patients with BRCA, CHOL, COAD, DLBC, HNSC, LGG, SKCM, STAD, and TGCT (**Figure 6C**; *P* < 0.05 for all). In addition, using the IMvigor210 (anti-PD-L1), GSE78220 (anti-PD-1), and GSE91061 (anti-CTLA4 and anti-PD-1) cohorts, we investigated the prognostic role of PTPN1 in patients who received ICI therapy. The OS of patients receiving ICI therapy for urinary system tumors in the IMvigor210 cohort with high PTPN1 expression was significantly longer than that of patients with low PTPN1 expression (**Figure 6D**). A similar result was obtained in the GSE78220 cohort (**Figure 6D**). In the GSE91061 cohort, melanoma patients with low PTPN1 expression showed better OS than those with high PTPN1 expression (**Figure 6D**). Taken together, these findings suggested that PTPN1 may regulate the activity of immune checkpoint genes, affecting immune cell infiltration, and may be a promising target for immunotherapy.

### PTPN1 was associated with immune infiltration and immune checkpoints in breast cancer

**Figure 7:**
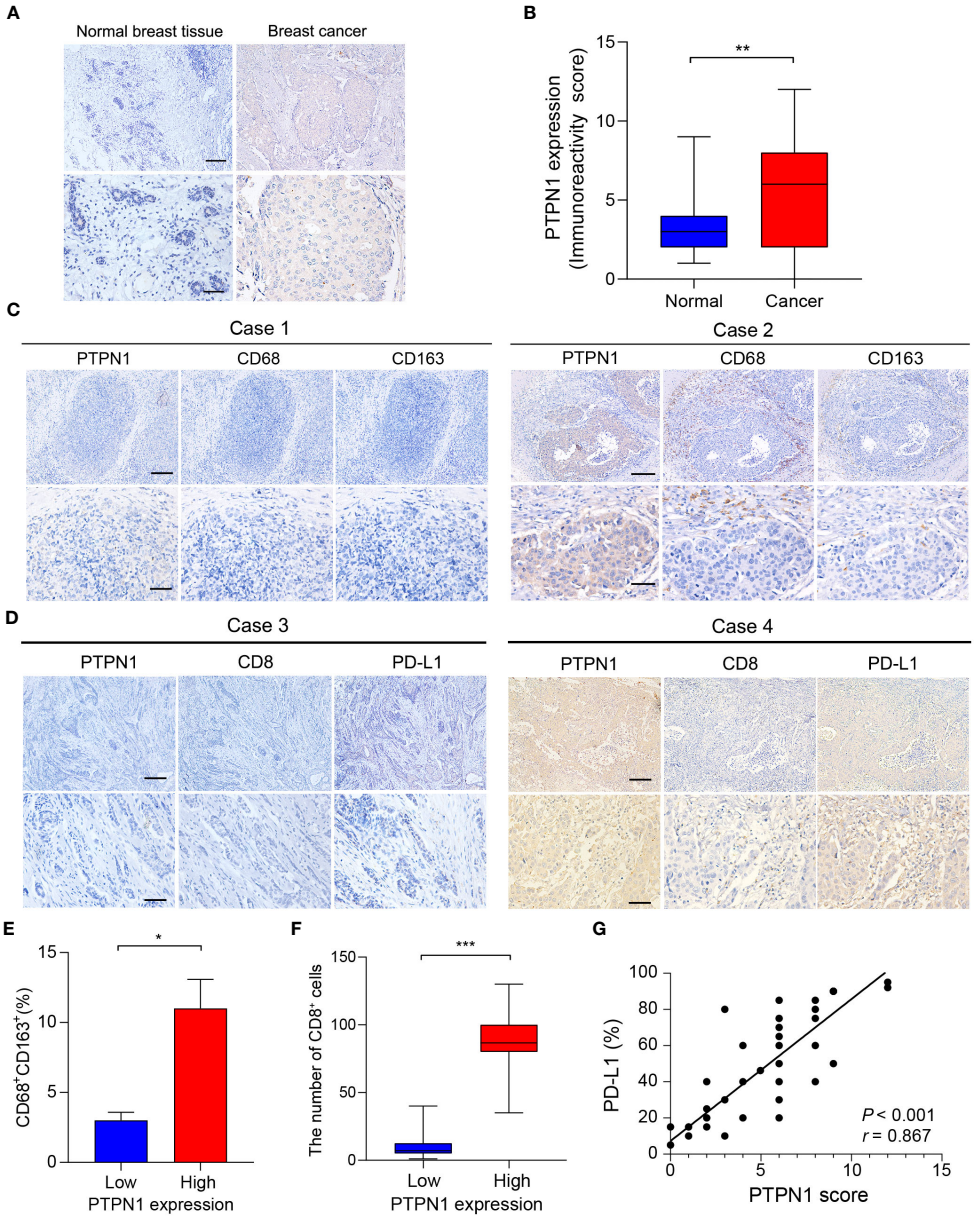
PTPN1 expression correlated with immune infiltration in human breast cancer tissues. **(A)** Representative PTPN1 staining in human breast cancer tissues and adjacent normal tissues (scale bars: top, 200 μm; bottom, 50 μm). **(B)** The immunoreactivity scores of PTPN1 in normal breast tissues and breast cancer tissues were compared using the Mann-Whitney U test (**P < 0.01). **(C)** Representative IHC staining images for PTPN1, CD68, and CD163 expression in three serial sections of the same tumor from two primary human breast cancer specimens (scale bars: top, 200 μm; bottom, 50 μm). **(D)** Representative IHC staining images for PTPN1, CD8, and PD-L1 expression in three serial sections of the same tumor from two primary human breast cancer specimens (scale bars: top, 200 μm; bottom, 50 μm.). **(E)** Quantitative determination of CD68^+^ CD163^+^ cells in the PTPN1 high- and low-expression groups. **(F)** The number of CD8^+^ T cells in breast cancer tissues with high or low PTPN1 expression. **(G)** The correlation between the expression levels of PTPN1 and PD-L1 proteins was evaluated using Pearson’s correlation. Error bars, SEM. *P < 0.05, ***P < 0.001 using Student’s t-test.

To further verify the expression of PTPN1 in breast cancer, IHC analysis was performed to detect the expression level of PTPN1 in 60 randomly selected tumor tissues and paired adjacent non-tumor tissues from patients with breast cancer. The results revealed that PTPN1 expression was significantly higher in tumor tissues than in matched non-tumor adjacent tissues and that PTPN1 was localized in the cytoplasm (**Figures 7A, B**). To validate the correlation of PTPN1 expression with immune cell infiltration, we analyzed the infiltration of TAMs and CD8^+^ T cells by performing IHC staining in serial sections of human breast cancer specimens from the same patient. The percentage of CD163^+^ M2-like TAMs in patients with high PTPN1 expression was significantly higher than that in patients with low PTPN1 expression (*P* = 0.02; **Figures 7C, E**). The number of CD8^+^ lymphocyte-infiltrating tissues in patients with high PTPN1 expression was significantly higher than that in patients with low PTPN1 expression (*P* < 0.001; **Figures 7D, F**). An increasing body of evidence suggests that immune checkpoint proteins, especially PD-L1, play a crucial role in anti-tumor immunotherapy. Hence, we evaluated PD-L1 expression in serial sections of the human breast cancer specimens from the same patient. The result showed that the PD-L1 protein level correlated positively with the PTPN1 expression level (*P* < 0.001; **Figures 7D, G**).

**Figure 8:**
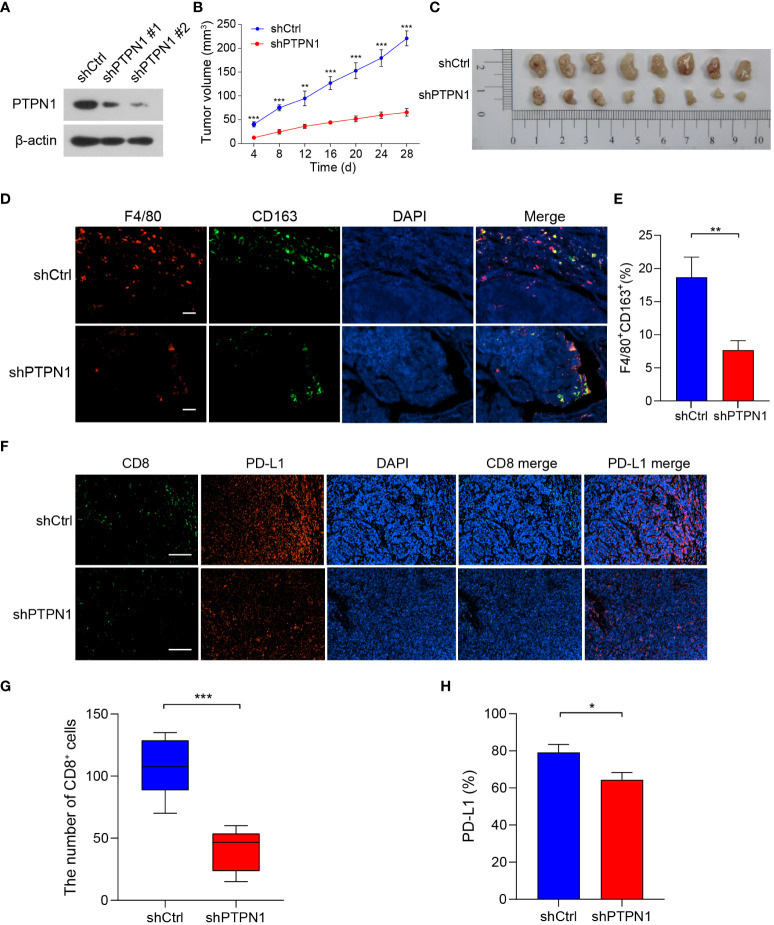
PTPN1 expression correlated with tumor growth and tumor immune infiltration *in vivo*. **(A)** PTPN1 knockdown in 4T1 cells was confirmed using immunoblotting. β-actin was used as the internal loading control. **(B)** 4T1-shPTPN1 and 4T1-shCtrl cells were subcutaneously injected into BALB/c mice, and tumor growth was monitored for 28 days. **(C)** Representative images of tumors derived from mice. **(D)** Representative images of immunofluorescence staining of CD163 (green) and F4/80 (red) in BALB/c mouse tumor tissues from 4T1-shPTPN1 and 4T1-shCtrl cells (scale bars: 100 μm). **(E)** Quantitative determination of F4/80^+^CD163^+^ cells in tumor tissues of 4T1-shPTPN1 versus 4T1-shCtrl cells. **(F)** Representative immunofluorescence images of CD8 (green) and PD-L1 (red) expression in BALB/c mouse tumor tissues from 4T1-shPTPN1 and 4T1-shCtrl cells (scale bars: 200 μm). **(G)** The number of CD8^+^ T cells in tumor tissues of 4T1-shPTPN1 versus 4T1-shCtrl cells. **(H)** Quantitative determination of PD-L1 protein expression in tumor tissues of 4T1-shPTPN1 versus 4T1-shCtrl cells. Error bars, SEM. ^*^ *P* < 0.05, ^**^ *P* < 0.01, ^***^ *P* < 0.001 using Student’s t-test.

To further validate the role of PTPN1 in the immune microenvironment of breast cancer *in vivo*, we knocked down the expression of PTPN1 in 4T1 breast cancer cells using two different short hairpin RNAs (shRNAs; shPTPN1#1 and shPTPN1#2), and a non-target shRNA as a control (shCtrl) (**Figure 8A**). The immunoblotting results showed successful knockdown of PTPN1 expression in 4T1 cells (**Figure 8A**). We selected shPTPN1#2 for subsequent experiments as its efficiency was higher than that of shPTPN1#1 (**Figure 8A**). Next, we used 4T1 cell lines with stable PTPN1 knockdown to perform *in vivo* mouse tumorigenesis assays. The results showed that tumors derived from knockdown of PTPN1 cells were significantly smaller than those from control cells (**Figures 8B, C**). Furthermore, we performed the immunofluorescent staining assay to analyze the correlation of PTPN1 expression with the infiltration of TAMs and CD8^+^ T cells and PD-L1 expression in the tumors derived from the PTPN1 knocked-down cells. PTPN1 deletion significantly diminished the percentage of CD163^+^ M2-like TAMs (*P* = 0.005; **Figures 8D, E**). Tumor infiltration of CD8^+^ T cells was significantly lower in tumors derived from the PTPN1 knocked-down cells than those derived from the control vector cells (**Figures 8F, G**). In addition, we observed that PTPN1 expression correlated positively with PD-L1 protein expression (**Figures 8F, H**). Collectively, these results suggested that PTPN1 was associated with immune infiltration and immune checkpoint gene expression.

### Correlation of PTPN1 expression with drug sensitivity in cancer cells

**Figure 9:**
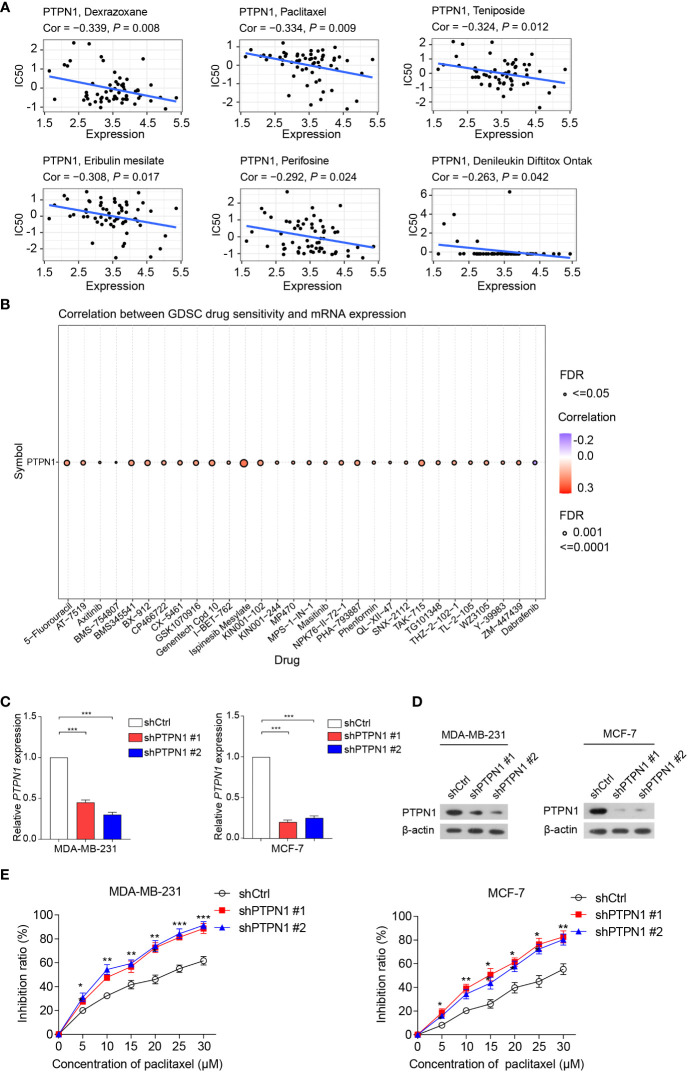
Correlation between PTPN1 level and drug sensitivity in cancer cells. **(A, B)** The correlation between PTPN1 expression and chemotherapeutic drug sensitivity in cancer cells was investigated using the data from the CellMiner **(A)** and GDSC databases **(B)**. **(C)** The mRNA level of *PTPN1* in MDA-MB-231 and MCF-7 cells with PTPN1 knockdown was examined using qRT-PCR. **(D)** The protein level of PTPN1 in MDA-MB-231 and MCF-7 cells with PTPN1 knockdown was examined using immunoblotting. β-actin was used as the internal loading control. **(E)** The cell inhibition ratios of MDA-MB-231 and MCF-7 cells with PTPN1 knockdown were evaluated using the CCK-8 viability assay. Cells were treated with various concentrations of paclitaxel (0, 5, 10, 15, 20, 25, and 30 µM) for 72 h. Data are shown as the means of three independent experiments. Error bars indicate SEM. ^*^ *P* < 0.05, ^**^ *P* < 0.01, ^***^ *P* < 0.001 using one-way ANOVA with *post hoc* intergroup comparisons.

Next, Pearson correlation analysis was performed to investigate the relationship between PTPN1 expression and drug sensitivity in cancer cells. The results showed that high PTPN1 expression was inversely related to the sensitivity of cancer cells to various chemotherapeutic drugs such as dexrazoxane, paclitaxel, teniposide, eribulin mesylate, perifosine, and denileukin diftitox (Ontak) (**Figure 9A**). In contrast, increased PTPN1 expression was strongly linked to increased sensitivity to various anticancer drugs, particularly ispinesib mesylate (**Figure 9B**). To further verify the relationship between PTPN1 and drug sensitivity in cancer cells, we performed the CCK-8 viability assay on breast cancer cell lines using various concentrations (0, 5, 10, 15, 20, 25, and 30 µM) of the chemotherapeutic drug, paclitaxel, which is widely used in breast cancer chemotherapy. We stably knocked down PTPN1 in the MDA-MB-231 and MCF-7 cells (**Figures 9C, D**) and found that PTPN1 depletion increased the sensitivity of these cells to paclitaxel (**Figure 9E**). These results suggested that PTPN1 plays a critical role in determining drug sensitivity.

### Construction of a PPI network for PTPN1 in cancers

To investigate the biological processes via which PTPN1 possibly regulates cancer progression, a PPI network was constructed using the GeneMANIA database. The results showed that PTPN1 interacted strongly with NOX4, JAK2, CTH, TRPV6, FCGR2A, and APBB1P (**Supplementary Figure S8A**). Furthermore, when subjected to GO analysis, the PPI network genes were found to be enriched in various GO terms, including peptidyl-tyrosine phosphorylation, regulation of tyrosine phosphorylation of STAT protein, tyrosine phosphorylation of STAT protein, to name a few (**Supplementary Figure S8B**). KEGG analysis revealed that the PPI network genes were enriched in multiple signaling pathways, including the JAK-STAT signaling pathway, PI3K-Akt signaling pathway, and PD-L1 expression and PD-1 checkpoint pathway in cancer (**Supplementary Figure S8C**). Based on the above results, we hypothesized that PTPN1 may contribute to tumor development and progression by regulating cancer-related processes or pathways.

## Discussion

In this study, we performed a comprehensive pan-cancer analysis of PTPN1 expression, integrating several databases (including TCGA, CCLE, TIMER, UALCAN, cBioPortal, and Kaplan-Meier Plotter). Moreover, we confirmed that high PTPN1 expression was closely associated with prognosis in most cancers. Furthermore, abnormal expression of PTPN1 was related to immune cell infiltration, TMB, MSI, and expression of immune checkpoint genes. In addition, the genes that interacted with PTPN1 were found to be mainly involved in tumorigenesis and regulation of immune response.

Recently, PTPN1 was found to play conflicting roles in the occurrence and development of various tumors (17). For example, in colorectal, prostate, gastric, and pancreatic cancers, PTPN1 may act as a tumor promoter (27–30). In contrast, PTPN1 was found to be a tumor suppressor in B cell lymphoma and esophageal cancer (31, 32). In this study, we found that PTPN1 expression was higher in BRCA, CHOL, COAD, ESCA, GBM, HNSC, KIRC, KIRP, LIHC, STAD, and THCA than in normal tissue, while it was lower in KICH, LUAD, LUSC, and THCA. Furthermore, our results showed that PTPN1 was highly expressed in many cancer cell lines. PTPN1 may play various roles in the prognosis of patients with various types of cancer. We found that PTPN1 overexpression was significantly associated with poor prognosis in ovarian (OS), lung (OS, FP, and PPS), and gastric cancers (OS). In contrast, increased PTPN1 expression was related to significantly better prognosis in breast (OS, DMSF, PPS, and RFS), ovarian (PFS), and gastric cancers (PPS). These results were consistent with those of previous studies. Reports have shown that high PTPN1 expression is associated with poor OS in patients with colorectal, pancreatic, and gastric cancers (27, 28, 30). However, high PTPN1 expression in hepatocellular carcinoma was significantly associated with good disease-free survival (DFS) and OS (33). Hence, these results suggest that PTPN1 may be a prognostic factor in different cancer types. Moreover, we performed a ROC curve analysis for KICH, UVM, LGG, LAML, MESO, and LIHC and found that PTPN1 had good predictive power for 1-, 3-, and 5-year OS. Furthermore, we observed that PTPN1 had good predictive efficacy for predicting 1-, 3-, and 5-year DSS in patients with KICH, UVM, LGG, GBM, BLCA, and BRCA. Our findings suggest that PTPN1 has a good predictive value for OS and DSS and may be useful for the prognostic evaluation of patients with multiple types of cancer. Emerging evidence suggests that the presence of tumor-infiltrating lymphocytes is important for predicting cancer progression in various solid tumors, as they affect cancer progression and response to therapy (34, 35). CD8^+^ T cells are important tumor-suppressing cells because they physically contact malignant tumor cells and induce tumor cell death by activating their intracellular signals (36, 37). Our results showed that PTPN1 was significantly positively correlated with the presence of CD8^+^ T cells in most cancer types. Previous results have indicated that the expression of PTPN1 was elevated in intratumoral CD8^+^ T cells, which inhibits the anti-tumor immunity of CD8^+^ T cells (20). PTPN1 is known to be associated with macrophage polarization (38). We found that PTPN1 expression showed a significant positive correlation with the presence of macrophages in most cancer types.

Immunotherapy, such as the use of ICIs, has revolutionized cancer treatment in recent years, demonstrating remarkable clinical benefits in patients with multiple cancers (39–41). Our findings indicated that PTPN1 expression was significantly associated with immune checkpoint gene expression and immune cell infiltration in patients with PAAD, LIHC, LUAD, OV, and UVM. Notably, in the IMvigor210, GSE78220, and GSE91061 cohorts, PTPN1 acted as an excellent biomarker for predicting prognosis and response to immunotherapy. A previous study has shown that targeting PTPN1 can improve T cell-mediated anti-tumor immunity and synergize with PD-1 checkpoint blockade and improve the efficacy of adoptively transferred chimeric antigen receptor (CAR) T cells (20). Furthermore, the genes that interacted with PTPN1 were found to be enriched in tumorigenesis-related and immune response pathways. Importantly, we further confirmed the abnormal expression of PTPN1 in breast cancer. Previous studies have shown that PTPN1 is associated with a significantly improved OS in breast cancer patients (42). We found that PTPN1 knockdown inhibited breast cancer cell tumorigenesis *in vivo*. Previous studies have also suggested that the PTPN1 protein level is dramatically increased in breast cancer tissues and that PTPN1 promotes the proliferation and suppresses the apoptosis of both ErbB2-positive and triple-negative breast cancer (TNBC) cell lines (43). Furthermore, PTPN1 deficiency delays ErbB2-induced mammary tumorigenesis and protects from lung metastasis. Hence, it was proposed that individuals with ErbB2-positive breast cancer might benefit from pharmacological inhibition of PTPN1 activity in combination with anti-ErbB2 therapies (44). We found that PTPN1 expression is associated with the extent of immune infiltration of CD163^+^ M2-like TAMs and CD8^+^ T cells and the expression of the immune checkpoint protein, PD-L1, in breast cancer. A recent study investigated the potentially novel physical interactions between the cytoplasmic domain of PD-L1 and PTPN1, which inhibits PTPN1 in TNBC. In this context, the PD-L1-mediated inhibition of PTPN1 in these TNBC cells possibly promotes tumorigenesis, which may recapitulate the tumor-suppressing role of PTPN1 in individuals who normally expresses high levels of PD-L1 (45). These findings suggest that PTPN1 may be used as a biomarker to predict immunotherapeutic responses in various cancers. However, our study has several limitations which warrant further investigations. First, the mechanism of PTPN1 alters immune cell infiltration levels in cancers requires further analysis using *in vivo* or *in vitro* experiments. In addition, we also identified some drugs specifically related to PTPN1. In particular, we found that PTPN1 deletion increased the sensitivity of MDA-MB-231 and MCF-7 cells to paclitaxel, the underlying mechanism of which remains unclear and requires investigation.

In conclusion, our findings suggest that PTPN1 may be a promising prognosis biomarker for multiple cancers and a predictive biomarker for immunotherapy. These findings add to our understanding of PTPN1’s potential role in tumor immunity and immunotherapy.

## Data availability statement

The original contributions presented in the study are included in the article/**Supplementary Material**. Further inquiries can be directed to the corresponding author.

## Ethics statement

The studies involving humans were approved by the Ethics Committee of The Third Hospital of Nanchang (IRB serial number: #K-ky2023049). The studies were conducted in accordance with the local legislation and institutional requirements. The participants provided their written informed consent to participate in this study. The animal study was approved by the Animal Care and Use Committee of Jinan University. The study was conducted in accordance with the local legislation and institutional requirements.

## Author contributions

RZ and HD conceived and designed the study. RZ, CX, SC and WC performed the data collection and analyses. SC, WC and HD performed the *in vitro* and *in vivo* experiments. RZ, HD, AZ and LY wrote and revised the manuscript. All authors contributed to the article and approved the submitted version.
