# Supplementary material for: PTPN1 is a prognostic biomarker related to cancer immunity and drug sensitivity: from pan-cancer analysis to validation in breast cancer
Source: Front Immunol. 2023 Oct 23;14:1232047. doi: 10.3389/fimmu.2023.1232047 (PMC10626546; doi:10.3389/fimmu.2023.1232047)
Supplement: Supplementary file 1 [file DataSheet_1.docx]

# Supplementary Information

**Supplementary Materials and Methods**

**Plasmid Constructs and Transfection**

Two independent PTPN1-targeting shRNA sequences and a scrambled sequence were inserted into the pLKO.1 lentiviral expression vector. The target sequences are as follows: human PTPN1 shRNA#1, 5′-TGCGACAGCTAGAATTGGAAA-3′; shRNA#2, 5′-GCTGCTCTGCTATATGCCTTA-3′; negative control scrambled sequence (shCtrl), 5'-TTCTCCGAACGTGTCACGT-3’. The MDA-MB-231, MCF-7, and 4T1 cells were transiently transfected using Lipofectamine 3000 (#L3000015, Thermo Fisher Scientific, Waltham, MA, USA) according to the manufacturer’s instructions.

**Xenograft Model**

Female BALB/c mice (5-week-old) were purchased from Vital River Lab Animal Technology Co. Ltd (Beijing, China). In brief, a suspension of 1 × 10^5^ 4T1 cells with stable knockdown of PTPN1 and control vector cells in 100 μL phosphate-buffered saline (PBS) were subcutaneously injected into the fourth mammary fat pad of virgin BALB/c mice (n = 8 in each group). Tumor size was measured using a caliper every 4 days after the appearance of palpable tumors, and the tumor volume was calculated using the formula: L× (S)^2^ × 0.52, where L and S are the long and short diameters of the tumors, respectively. The mice were euthanized 28 days after the formation of the palpable tumors. The tumors were excised, fixed in formalin, and embedded in paraffin. All the animal experiments were approved by the Animal Care and Use Committee of Jinan University.

**Supplementary Figures and Figure legends**


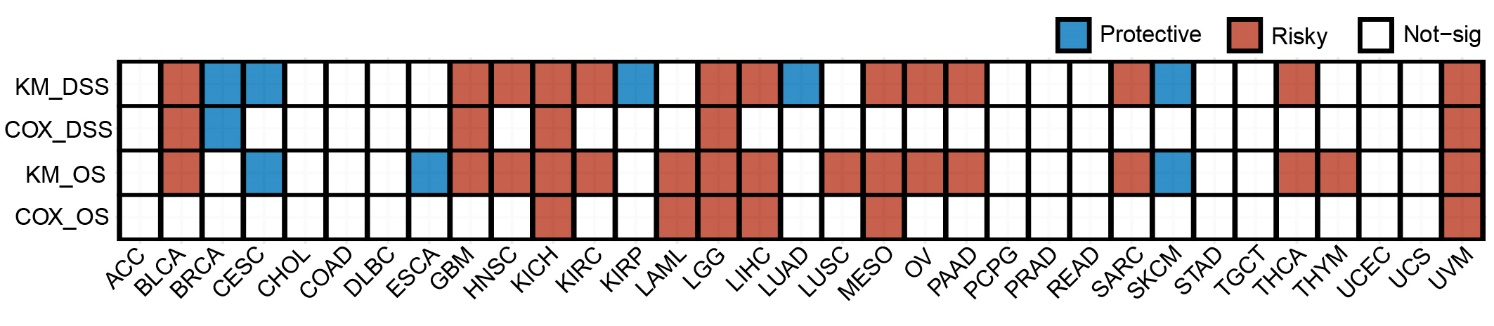


**Figure S1****.** The correlation between PTPN1 expression and OS, and DSS in the cancer types indicated was investigated using univariate Cox regression and Kaplan-Meier models. The color red indicates that PTPN1 is a risk factor for cancer patients’ prognosis, while the color blue represents a protective factor.


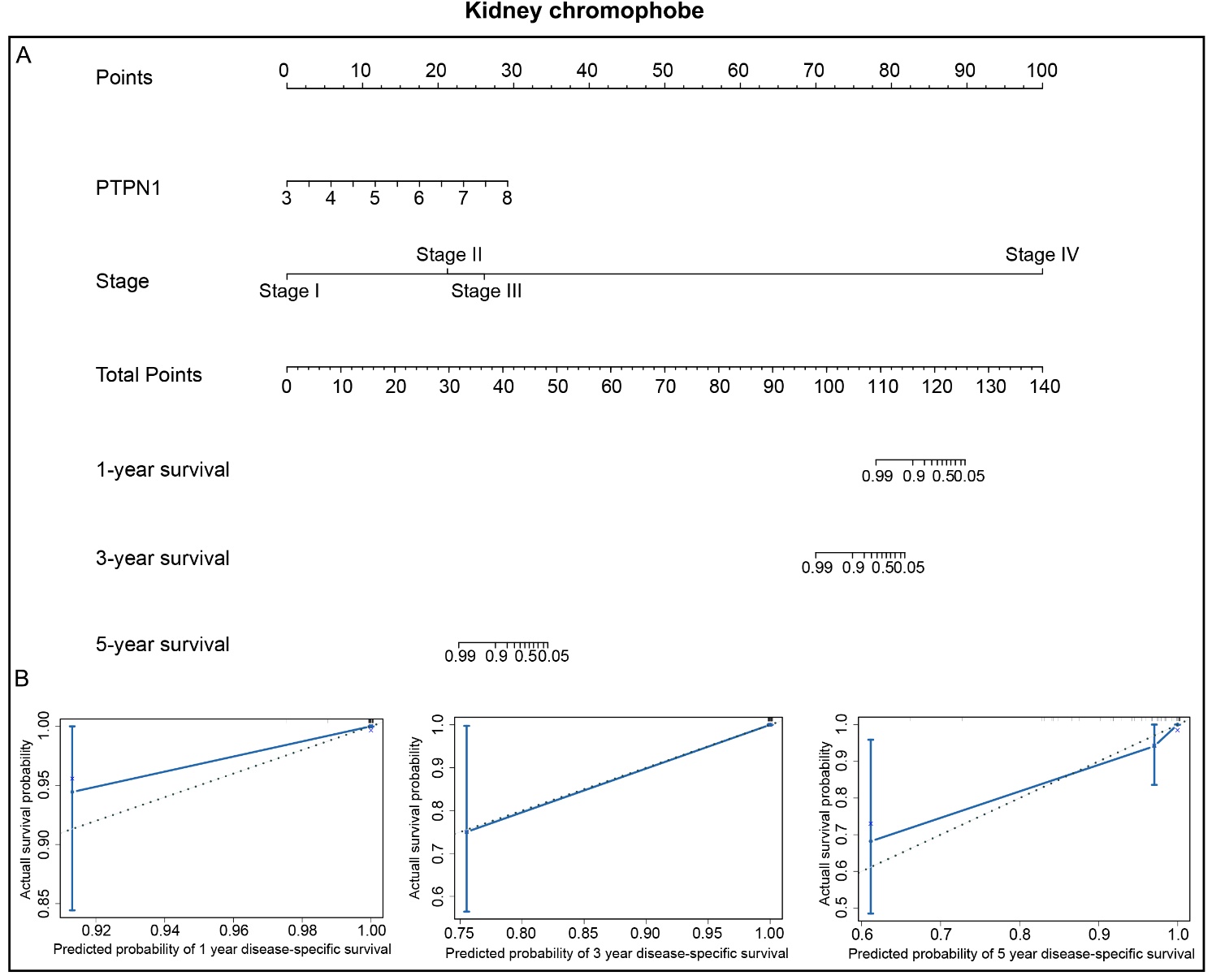
**Figure S2.** Nomogram for predicting the DSS probability in kidney chromophobe (KICH). (**A**) Nomogram analysis of PTPN1 expression and other clinical features in patients with KICH. (**B**) Calibration analysis of 1-year, 3-year and 5-year DSS of nomogram containing PTPN1 gene in patients with KICH.


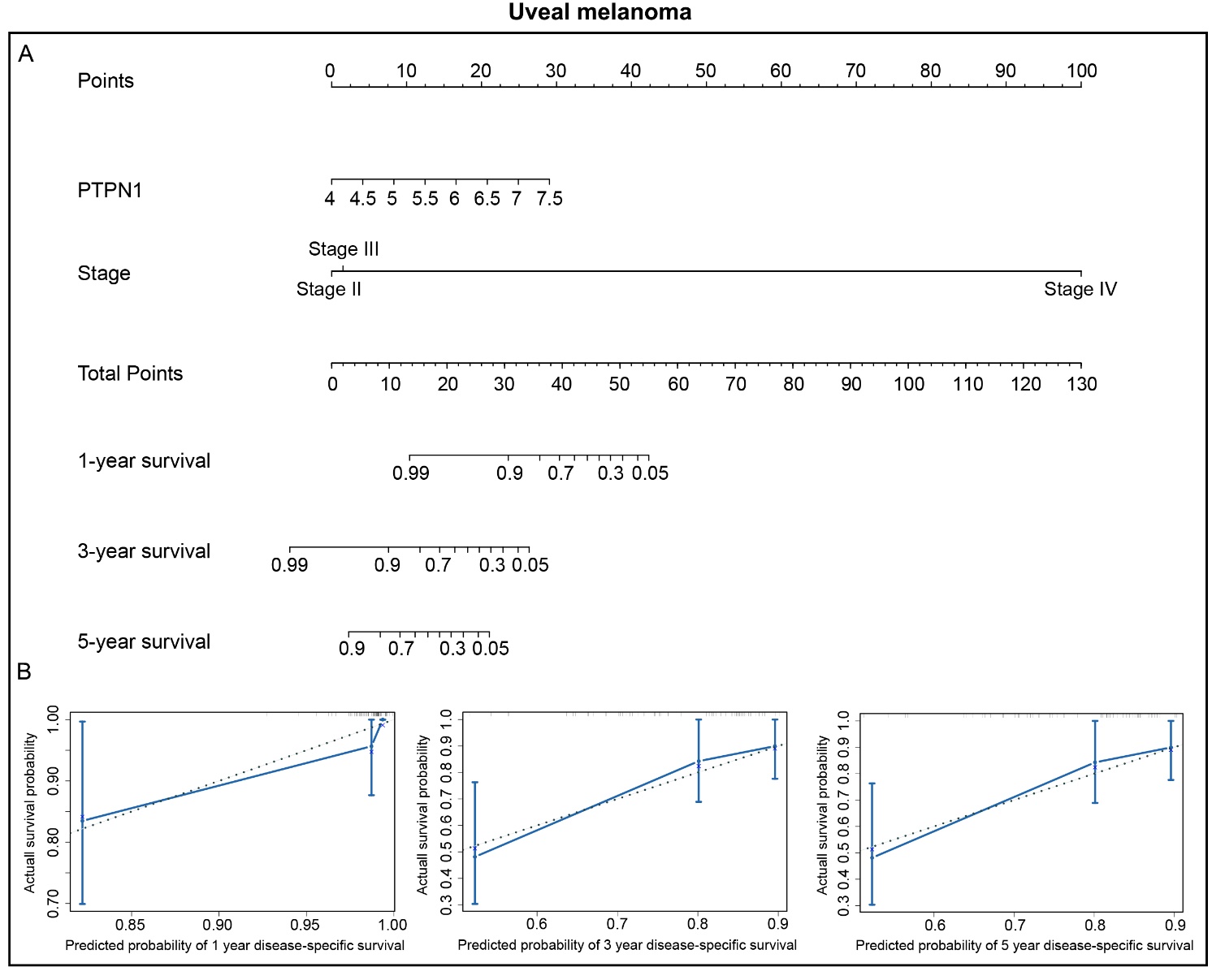
**Figure S3.** Nomogram for predicting the DSS probability in uveal melanoma (UVM). (**A**) Nomogram analysis of PTPN1 expression and other clinical features in patients with UVM. (**B**) Calibration analysis of 1-year, 3-year and 5-year DSS of nomogram containing PTPN1 gene in patients with UVM.


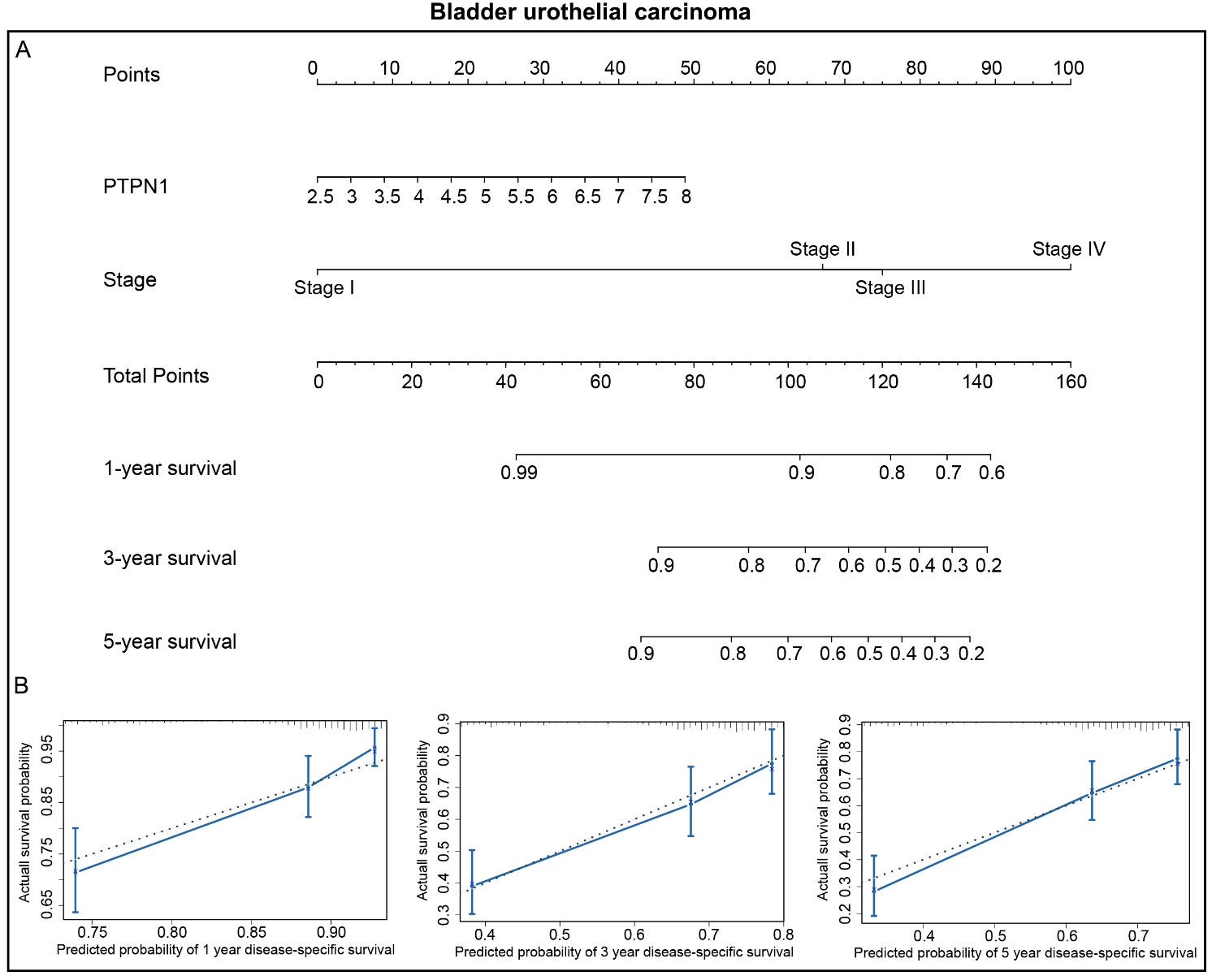
**Figure S4.** Nomogram for predicting the DSS probability in bladder urothelial carcinoma (BLCA). (**A**) Nomogram analysis of PTPN1 expression and other clinical features in patients with BLCA. (**B**) Calibration analysis of 1-year, 3-year and 5-year DSS of nomogram containing PTPN1 gene in patients with BLCA.


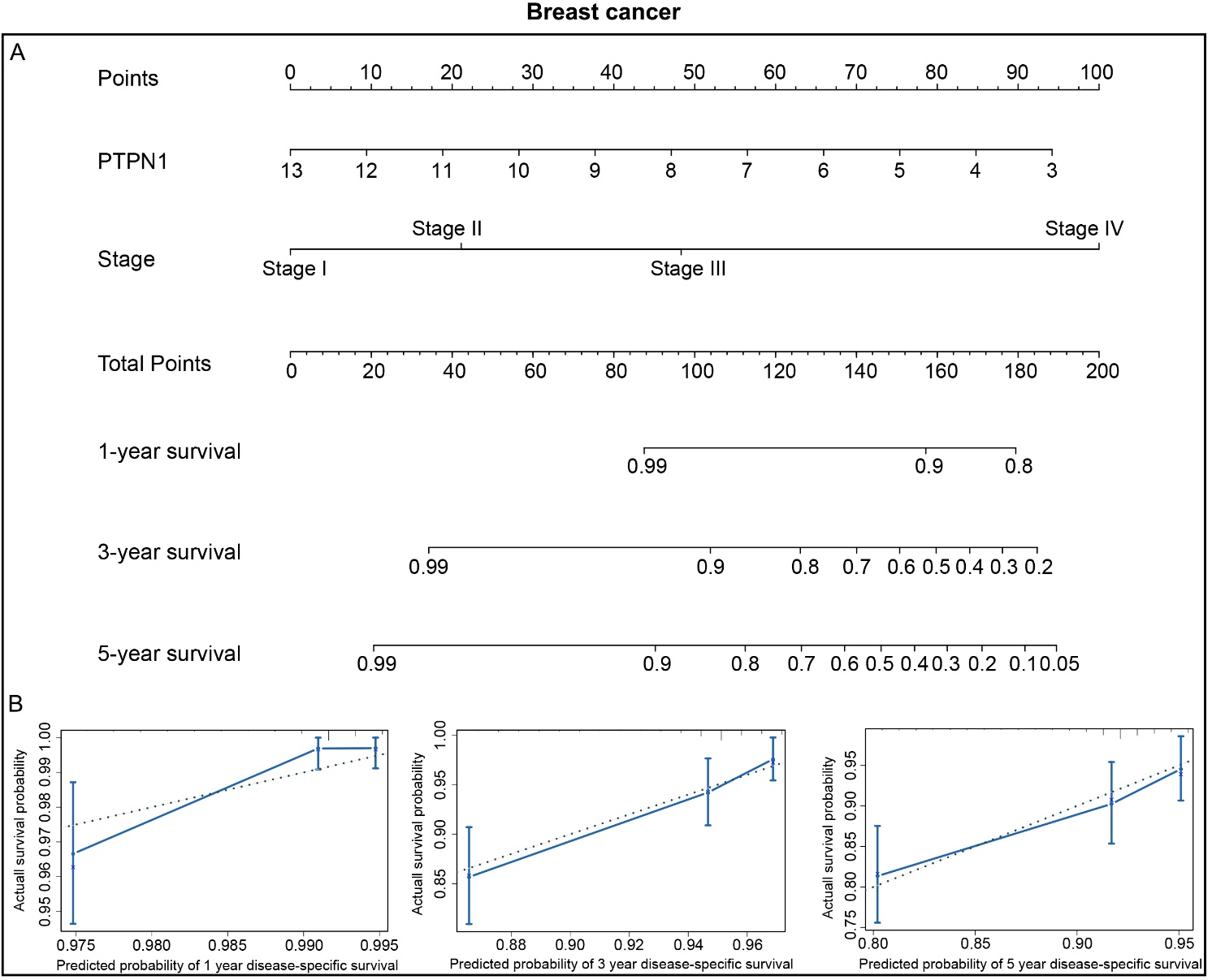
**Figure S5.** Nomogram for predicting the DSS probability in breast cancer (BRCA). (**A**) Nomogram analysis of PTPN1 expression and other clinical features in patients with BRCA. (**B**) Calibration analysis of 1-year, 3-year and 5-year DSS of nomogram containing PTPN1 gene in patients with BRCA.


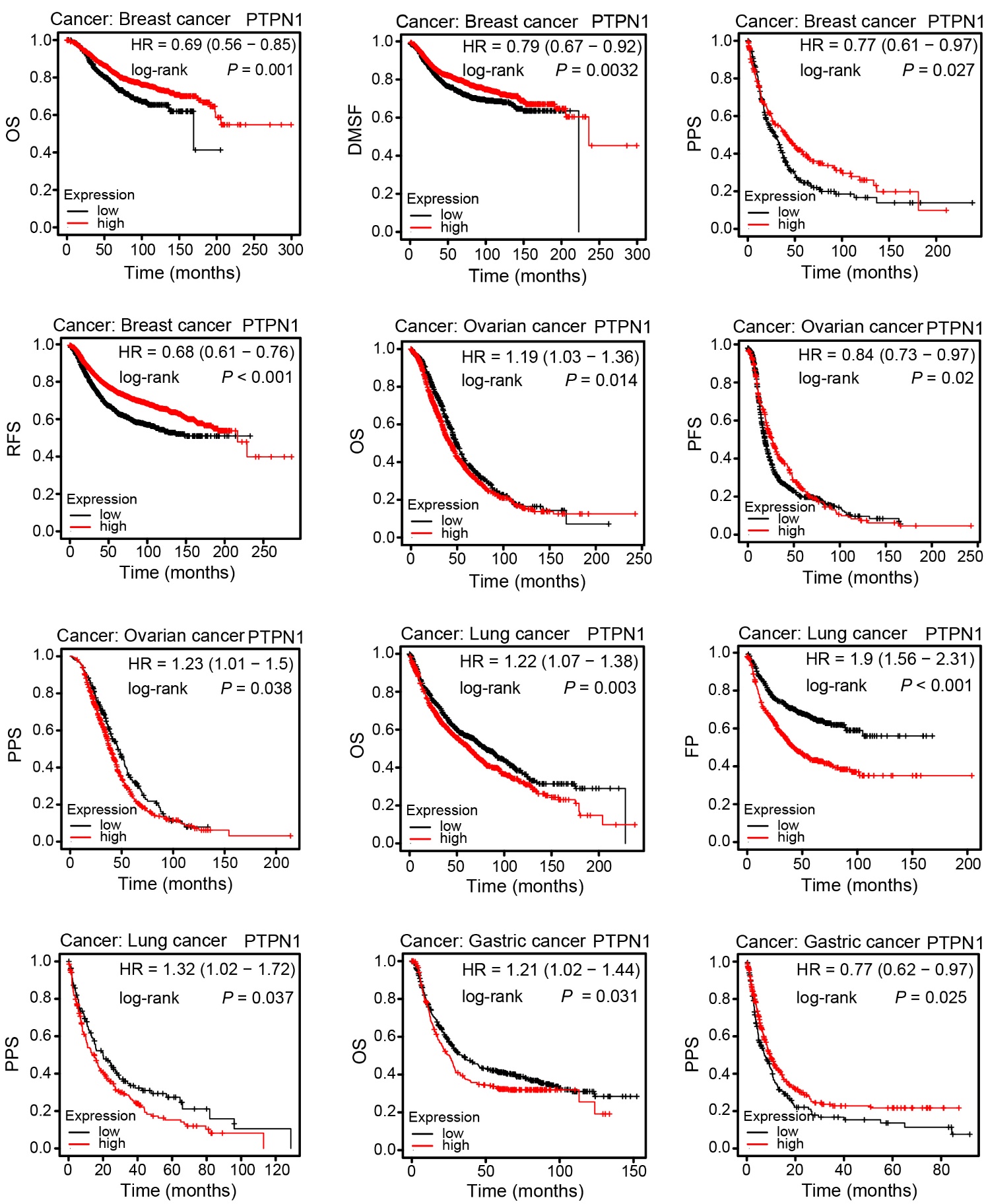


**Figure S6.** The prognosis value of PTPN1 in breast cancer (OS, DMSF, PPS, and RFS), ovarian (OS, PFS, and PPS), lung (OS, FP, and PPS), and gastric cancer (OS and PPS) was investigated in Kaplan-Meier Plotter database.


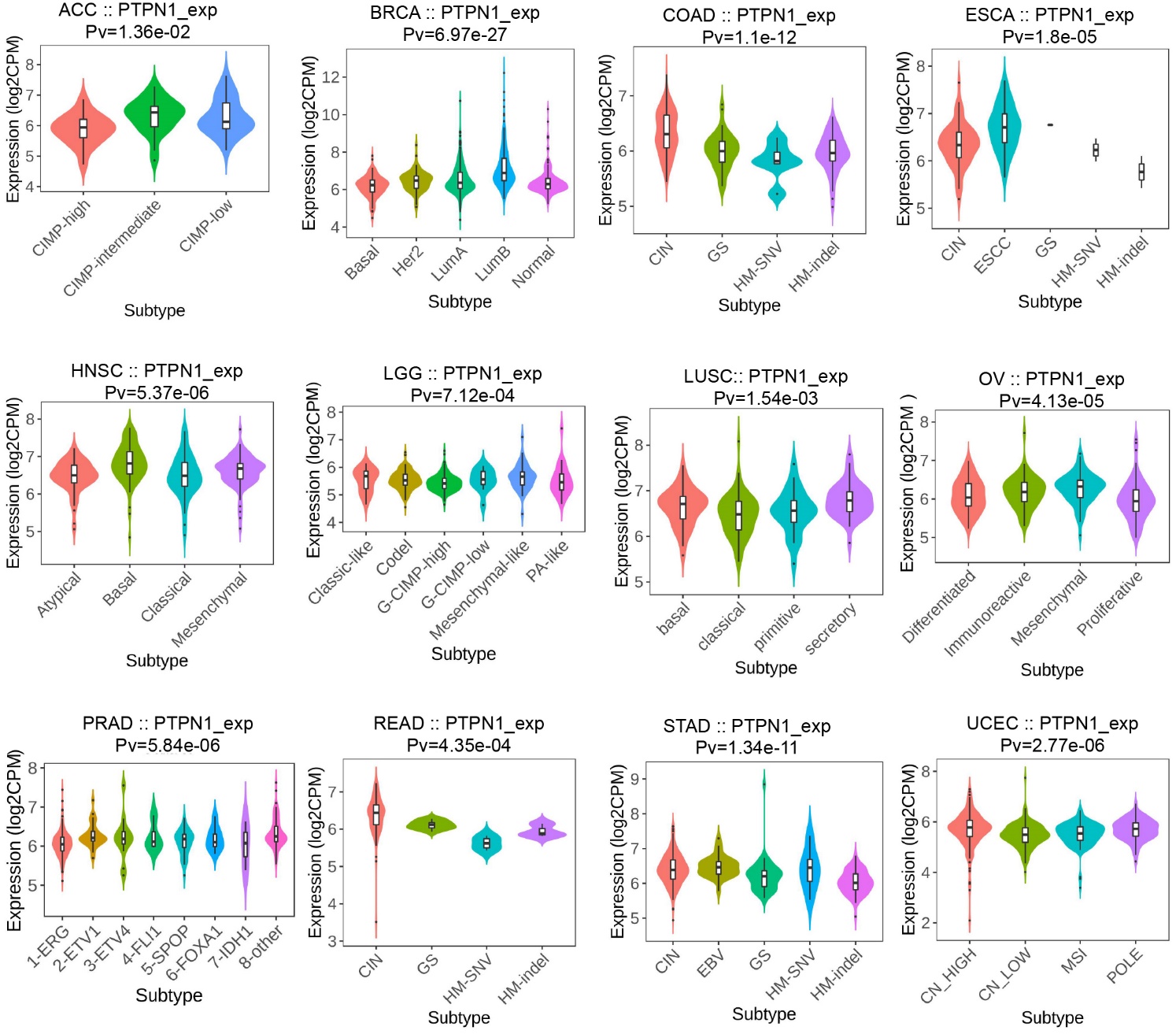


**Figure S7.** Relationship between PTPN1 expression and molecular subtypes in cancers.


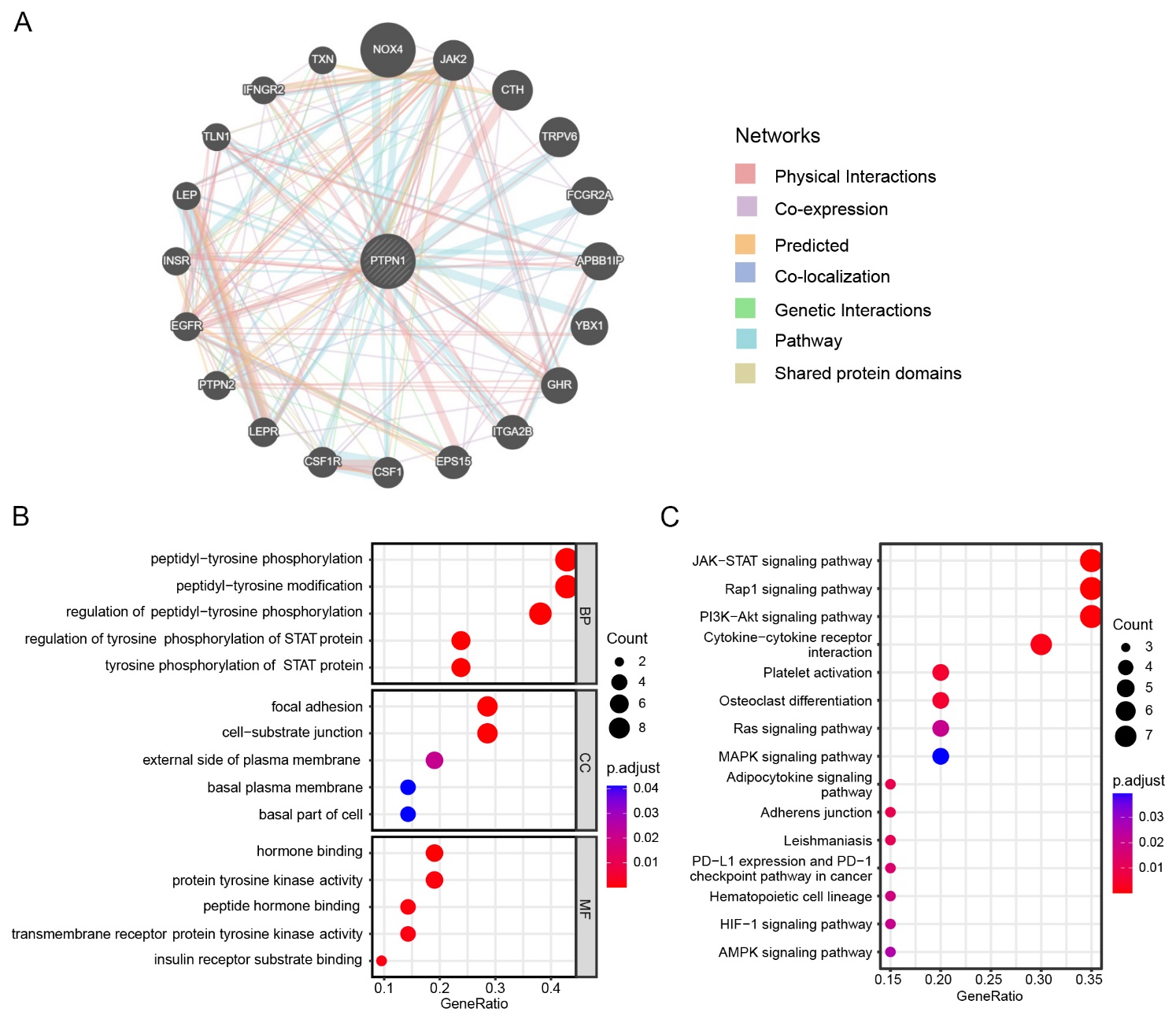


**Figure S8.** A PPI network for PTPN1 in cancers. (**A**) Construction of a PPI Network for PTPN1 in cancers. (**B**) GO and (**C**) KEGG analyses of the PPI-network genes.
